# Supplementary material for: Ontogenetic variability in crystallography and mosaicity of conodont apatite: implications for microstructure, palaeothermometry and geochemistry
Source: R Soc Open Sci. 2020 Jul 8;7(7):200322. doi: 10.1098/rsos.200322 (PMC7428274; doi:10.1098/rsos.200322)
Supplement: X-ray diffraction and statistical analysis [file rsos200322supp1.pdf]

Electronic supplementary information

Ontogenetic variability in crystallography and mosaicity of conodont apatite: Implications for microstructure, paleothermometry and geochemistry

Mohammad Shohel[1], Neo E.B. McAdams[2], Bradley D. Cramer*[3], Tori Z. Forbes*[1]

[1]Department of Chemistry, University of Iowa, Iowa City, IA 52242, USA

[2]Department of Geosciences, Texas Tech University, Lubbock, TX 79409, USA

[3]Department of Earth and Environmental Sciences, University of Iowa, Iowa City, IA 52242, USA

*Corresponding authors: tori-forbes@uiowa.edu; bradley-cramer@uiowa.edu

Table of Contents

1. **X-ray diffraction experiment**
2. **Structural information – Unit Cell parameters**
3. **Correlation between *c* and *a***

1. **X-ray diffraction experiment**

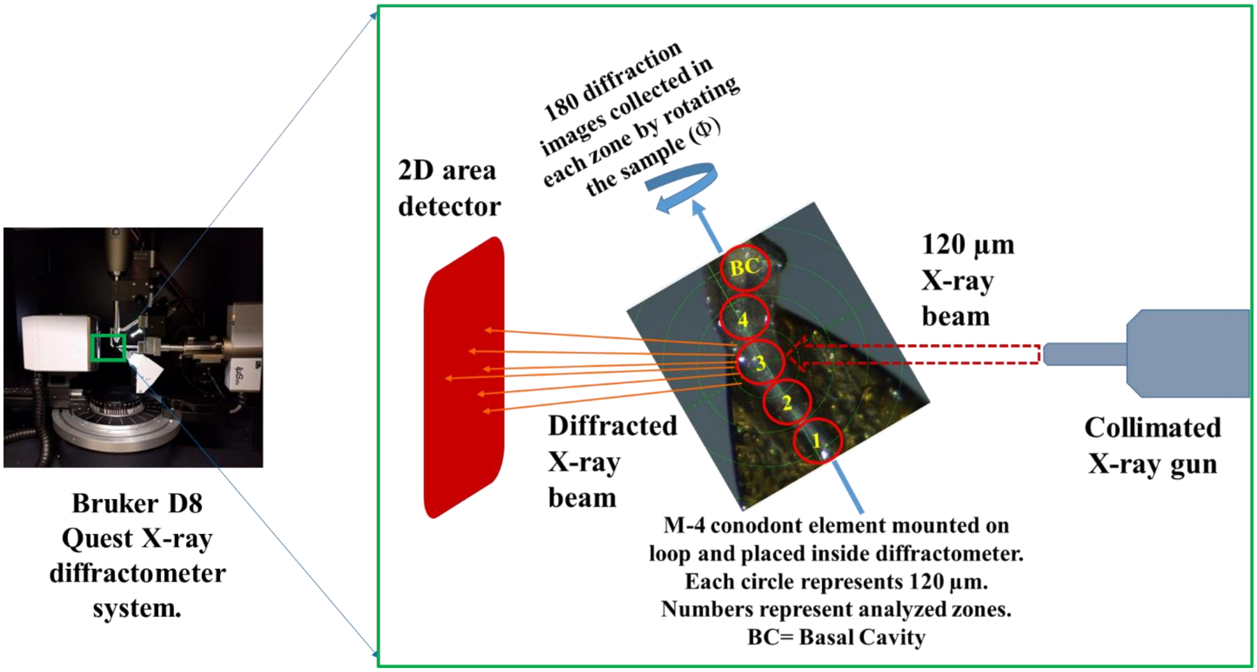

Figure S1. A schematic diagram of the X-ray diffraction experiment carried out on Bruker D8 Quest single crystal X-ray diffractometer system.

2. Structural Information – Unit Cell Parameters

Table S1. Unit cell parameters determined from highly crystalline reflections of S_a elements. Single-crystal X-ray analysis procedures were used to calculate unit cell parameters. NR (not refined) represents regions where single-crystal X-ray diffraction refinement methods could not be used to determine the unit cell parameters. Juvenile and older conodont samples are labeled as J and M, respectively.

**Region**	**Sample**	**Unit cell parameter**		**Volume**	**Mosaicity (°)**
		***a* (Å)**	***c* (Å)**	**($Å^3$)**	
1	S_a-1 (J)	9.371(10)	6.897(7)	524.6(11)	1.18
	S_a-2 (J)	NR	NR	NR	NR
	S_a-3 (J)	9.362 (8)	6.896 (6)	523.6(9)	1.14
	S_a-4 (O)	NR	NR	NR	NR
	S_a-5 (O)	NR	NR	NR	NR
2	S_a-1 (J)	9.361(13)	6.893(9)	523.1(14)	1.19
	S_a-2 (J)	9.377(6)	6.894(4)	525(6)	1.31
	S_a-3 (J)	9.340(20)	6.882(15)	520(2)	1.14
	S_a-4 (O)	NR	NR	NR	NR
	S_a-5 (O)	NR	NR	NR	NR
3	S_a-1 (J)	9.368(8)	6.899(6)	524.4(8)	1.24
	S_a-2 (J)	9.386(5)	6.902(3)	526.6(5)	1.32
	S_a-3 (J)	NA	NA	NA	NA
	S_a-4 (O)	9.326(3)	6.864(8)	517.0(13)	1.35
	S_a-5 (O)	NR	NR	NR	NR
4	S_a-1 (J)	NA	NA	NA	NA
	S_a-2 (J)	NA	NA	NA	NA
	S_a-3 (J)	NA	NA	NA	NA
	S_a-4 (O)	9.384(6)	6.900(4)	526.2(6)	1.33
	S_a-5 (O)	9.395(6)	6.911(3)	528.3(6)	1.33

NA = Not analyzed.

*numbers in parenthesis () mean standard deviation

Table S2. Unit cell parameters determined from highly crystalline reflections of S_{b-c} elements. Single-crystal X-ray analysis procedures were used to calculate unit cell parameters. NR (not refined) represents regions where single-crystal X-ray diffraction refinement methods could not be used to determine the unit cell parameters. Juvenile and older conodont samples are labeled as J and M, respectively.

**Region**	**Sample**	**Unit cell parameter**		**Volume**	**Mosaicity (°)**
		a (Å)	c (Å)	**(Å^3)**	
1	S_{b-c}-1 (J)	9.363(6)	6.903(5)	524.1(17)	1.21
	S_{b-c}-2 (J)	9.378(11)	6.912(11)	526.5(11)	1.17
	S_{b-c}-3 (O)	NR	NR	NR	NR
	S_{b-c}-4 (M)	9.390(60)	6.880(30)	532(6)	1.22
	S_{b-c}-5 (O)	9.371(10)	6.897(7)	524.6(11)	1.18
2	S_{b-c}-1 (J)	NR	NR	NR	NR
	S_{b-c}-2 (J)	9.372(5)	6.907(3)	525.4(5)	1.27
	S_{b-c}-3 (O)	NR	NR	NR	NR
	S_{b-c}-4 (O)	9.361(11)	6.880(7)	522.1(11)	1.27
	S_{b-c}-5 (O)	NR	NR	NR	NR
3	S_{b-c}-1 (J)	NA	NA	NA	NA
	S_{b-c}-2 (J)	NA	NA	NA	NA
	S_{b-c}-3 (O)	9.380(50)	6.890(30)	525(5)	1.28
	S_{b-c}-4 (O)	9.390(10)	6.905(6)	527.2(10)	1.29
	S_{b-c}-5 (O)	NR	NR	NR	NR
4	S_{b-c}-1 (J)	NA	NA	NA	NA
	S_{b-c}-2 (J)	NA	NA	NA	NA
	S_{b-c}-3 (O)	NR	NR	NR	NR
	S_{b-c}-4 (O)	9.380(6)	6.899(4)	525.7(6)	1.29
	S_{b-c}-5 (O)	9.378(7)	6.886(5)	524.4(8)	1.33
5	S_{b-c}-1 (J)	NA	NA	NA	NA
	S_{b-c}-2 (J)	NA	NA	NA	NA
	S_{b-c}-3 (O)	9.375(15)	6.883(10)	523.8(16)	1.31
	S_{b-c}-4 (O)	NR	NR	NR	NR
	S_{b-c}-5 (O)	9.383(7)	6.890(4)	525.3(7)	1.35

NA = Not analyzed.

*numbers in parenthesis () mean standard deviation

Table S3. Unit cell parameters determined from highly crystalline reflections of M elements. Single-crystal X-ray analysis procedures were used to calculate unit cell parameters. Juvenile and older conodont samples are labeled as J and M, respectively.

**Region**	**Sample**	**Unit cell parameter**		**Volume**	**Mosaicity**
		a (Å)	c (Å)	**(Å^3)**	
1	M-1 (J)	9.370(80)	6.910(60)	526(9)	1.15
	M-2 (J)	9.400(6)	6.910(4)	528.8(6)	1.08
	M-3 (O)	9.330(40)	6.850(30)	516(4)	1.25
	M-4 (O)	9.371(8)	6.887(5)	523.8(8)	1.29
2	M-1 (J)	9.330(20)	6.855(15)	517.(2)	1.28
	M-2 (J)	9.388(6)	6.889(4)	526.6(7)	1.17
	M-3 (O)	9.373(12)	6.882(8)	526.3(13)	1.31
	M-4 (O)	9.350(20)	6.876(16)	521(3)	1.31
3	M-1 (J)	9.379(5)	6.896(6)	525.4(5)	1.30
	M-2 (J)	NA	NA	NA	NA
	M-3 (O)	9.380(30)	6.885(17)	525(3)	1.30
	M-4 (O)	9.383(7)	6.895(5)	525.8(7)	1.36
4	M-1 (J)	NA	NA	NA	NA
	M-2 (J)	NA	NA	NA	NA
	M-3 (O)	9.369(5)	6.881(3)	523.0(5)	1.32
	M-4 (O)	9.387(4)	6.898(3)	526.3(4)	1.38

NA = Not analyzed.

*numbers in parenthesis () mean standard deviation

Table S4. Unit cell parameters from natural apatite crystals. The Durango and volcanic apatite samples measured by this study are plotted in text figure 4.

Sample	Unit cell parameter		Volume	Mosaicity (°)
	a (Å)	c (Å)	(Å^3)	
**Durango apatite (this study)**	9.4098(7)	6.8869(6)	528.10(8)	0.96
**Volcanic apatite (this study)**	9.4287(8)	6.8666(6)	528.65(7)	0.95
**Fluorapatite (Durango, Mexico)[1]**	9.398(3)	6.878(2)	-	-
**Hydroxyapatite (Holly Springs, Georgia)[1]**	9.418(2)	6.875(2)	-	-
**Chlorapatite (Kragero, Norway)[1]**	9.598(2)	6.776(4)	-	-

*numbers in parenthesis () mean standard deviation

Table S5. Unit cell parameters determined from the Debye rings within the polycrystalline region of the basal cavity of conodont elements. Juvenile and mature conodont samples are labeled as J and M, respectively.

**Sample**	**Unit cell parameter**	
	a (Å)	c (Å)
Sa-1 (J)	9.3915(28)	6.8276(27)
Sa-2 (J)	9.3819(17)	6.8805(17)
Sa-3 (J)	9.3915(28)	6.8276(27)
Sa-4 (O)	9.3616(23)	6.8606(22)
Sa-5 (O)	9.3540(14)	6.8629(14)
Sb-c-1 (J)	9.3758(12)	6.8923(12)
Sb-c-2 (J)	9.4242(25)	6.8622(25)
Sb-c-3 (O)	9.3882(24)	6.8889(24)
Sb-c-4 (O)	9.3667(16)	6.8939(16)
Sb-c-5 (O)	9.3923(22)	6.8790(22)
M-1 (J)	9.3863(19)	6.8842(19)
M-2 (J)	9.3880(12)	6.8788(12)
M-3 (O)	9.4016 (21)	6.8869(21)
M-4 (O)	9.3862(23)	6.8559(22)

*numbers in parenthesis () mean standard deviation

3. Correlation between *c* and *a*

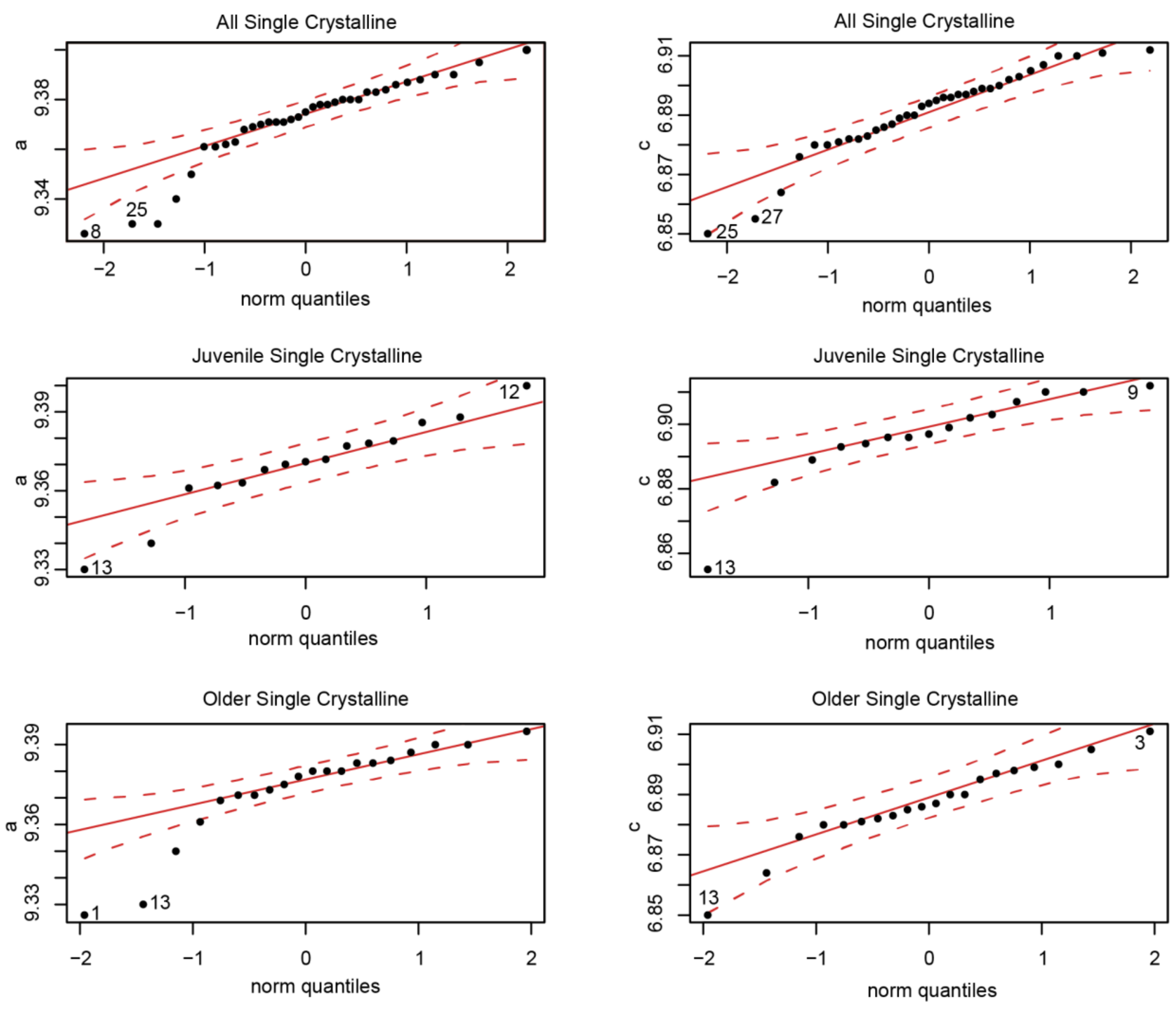

Figure S2. QQ Plots for all single crystalline zones to test for normal distribution. Plots separated by axis (*a*, *c*), and ontogenetic grouping (all, juvenile, older).

Table S6. Summary results of model II linear regressions performed in 'R' utilizing the "lmodel2" package [2]. Data table utilized for "lmodel2" regression analyses is included as a .csv file in the electronic supplement (Shohel et al – All Data for lmodel2 Runs.csv). Program calls are provided at the bottom of the supplementary file. The correlation coefficient (r), coefficient of determination (r-squared), and two-tailed parametric p-values of the model II Ordinary Least Squares (OLS) regressions are provided. Ordinary Least Squares (OLS), Major-Axis (MA), Standard Major-Axis (SMA), and Ranged Major Axis (RMA) regressions with the one-tailed permutation test p-value (n=10,000), in the direction of the sign of the slope, returned from the lmodel2 runs for each group of samples are also provided. The permutation test for SMA is not applicable (NA) because the SMA slope cannot be tested [2]. The regressions are neither significant nor valid for the polycrystalline samples due to the extremely small size of the total population of samples.

All Single Crystalline

r	0.7302493
r-squared	0.533264
two-tailed parametric p-value	***6.38724E-07***
Regression Type	*one-tailed permutation test p-value (n=10,000) in the direction of the sign of the slope*
OLS	***0.0001***

All Polycrystalline

r	-0.1337636
r-squared	0.01789271
two-tailed parametric p-value	0.6484671
Regression Type	*one-tailed permutation test p-value (n=10,000) in the direction of the sign of the slope*
OLS	0.3171

MA	***0.0001***
SMA	NA
RMA	***0.0001***

MA	0.3171
SMA	NA
RMA	0.3171

Juvenile Single Crystalline

r	0.7314171
r-squared	0.5349709
two-tailed parametric p-value	***0.001943149***
Regression Type	*one-tailed permutation test p-value (n=10,000) in the direction of the sign of the slope*
OLS	***0.0034***
MA	***0.0034***
SMA	NA
RMA	***0.0034***

Juvenile Polycrystalline

r	-0.3258405
r-squared	0.106172
two-tailed parametric p-value	0.4757275
Regression Type	*one-tailed permutation test p-value (n=10,000) in the direction of the sign of the slope*
OLS	0.2820
MA	0.2820
SMA	NA
RMA	0.2820

Older Single Crystalline

r	0.8567035
r-squared	0.7339409
two-tailed parametric p-value	1.42126E-06
Regression Type	*one-tailed permutation test p-value (n=10,000) in the direction of the sign of the slope*
OLS	***0.0001***
MA	***0.0001***
SMA	NA
RMA	***0.0001***

Older Polycrystalline

r	0.3851273
r-squared	0.1483231
two-tailed parametric p-value	0.3935765
Regression Type	*one-tailed permutation test p-value (n=10,000) in the direction of the sign of the slope*
OLS	0.1971
MA	0.1971
SMA	NA
RMA	0.1971

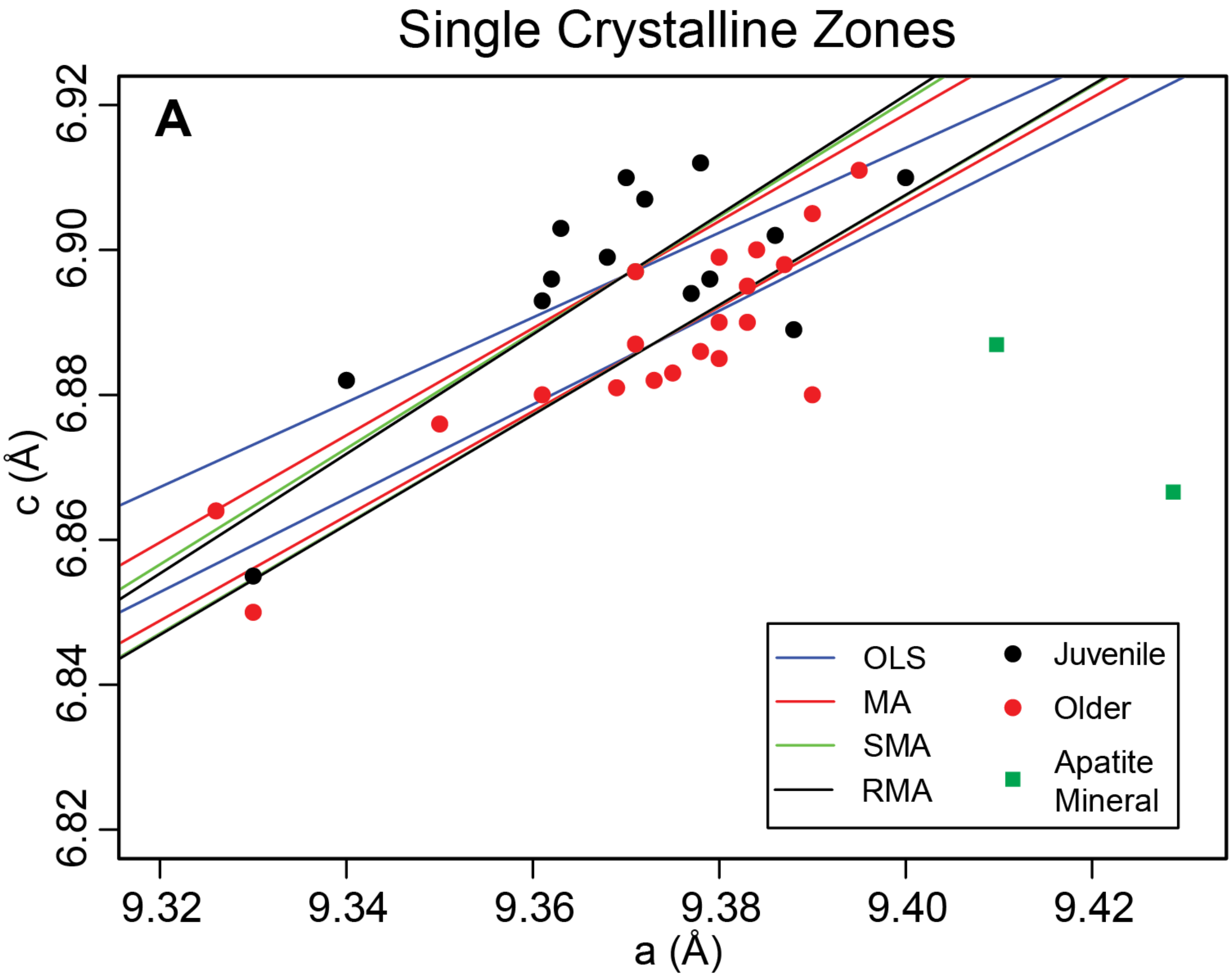

Figure S3. Same as Text Figure 4 Panel A, but with regression lines for all four model II regression methods (OLS, MA, SMA, and RMA) for each set of data (juvenile and older specimens).

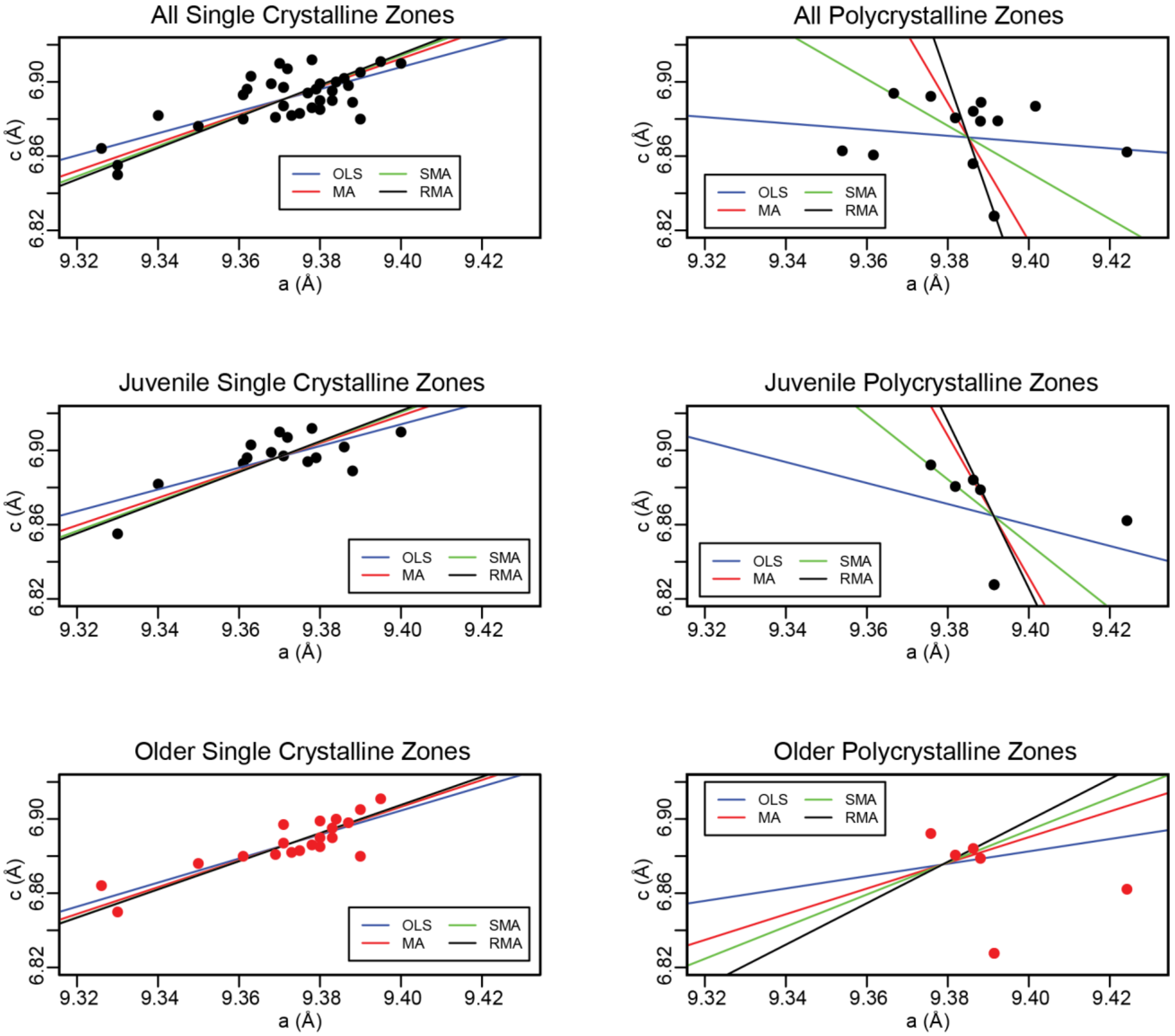

Figure S4. All populations separated with regression analyses performed for each population. Each image shows regression lines for all four model II regression methods (OLS, MA, SMA, and RMA).

Table S7. Complete model II regression results for single crystalline zone

<u>Model II Regression (Juvenile Single Crystalline Zones)</u>

Call: lmodel2(formula = data$cj ~ data$aj, data = data, range.y = "relative", range.x = "relative", nperm = 10000)

n = 15   r = 0.7314171   r-square = 0.5349709

Parametric P-values: 2-tailed = 0.001943149   1-tailed = 0.0009715747

Angle between the two OLS regression lines = 17.23159 degrees

Permutation tests of OLS, MA, RMA slopes: 1-tailed, tail corresponding to sign

A permutation test of r is equivalent to a permutation test of the OLS slope

P-perm for SMA = NA because the SMA slope cannot be tested

Regression results

Method	Intercept	Slope	Angle (degrees)	P-perm (1-tailed)
OLS	1.41272985	0.5852506	30.33833	0.00339966
MA	***-0.02618356***	***0.7388221***	***36.45781***	***0.00339966***
SMA	-0.60089888	0.8001600	38.66540	NA
RMA	-0.83627935	0.8252815	39.53223	0.00339966

Confidence intervals

Method	2.5%-Intercept	97.5%-Intercept	2.5%-Slope	97.5%-Slope
OLS	-1.650629	4.476089	0.2583069	0.9121944
MA	***-5.071851***	***3.304844***	***0.3833103***	***1.2773330***
SMA	-4.265949	1.860760	0.5374336	1.1913211
RMA	-7.489006	2.559339	0.4628760	1.5353096

Eigenvalues: 0.0004476579 6.496116e-05

H statistic used for computing C.I. of MA: 0.07128577

<u>Model II Regression (Older Single Crystalline Zones)</u>

Call: lmodel2(formula = data$cm ~ data$am, data = data, range.y = "relative", range.x = "relative", nperm = 10000)

n = 20   r = 0.8567035   r-square = 0.7339409

Parametric P-values: 2-tailed = 1.421256e-06   1-tailed = 7.10628e-07

Angle between the two OLS regression lines = 8.495407 degrees

Permutation tests of OLS, MA, RMA slopes: 1-tailed, tail corresponding to sign

A permutation test of r is equivalent to a permutation test of the OLS slope

P-perm for SMA = NA because the SMA slope cannot be tested

Regression results

Method	Intercept	Slope	Angle (degrees)	P-perm (1-tailed)
OLS	0.8208079	0.6472070	32.91123	9.999e-05
MA	***0.1205420***	***0.7219196***	***35.82626***	***9.999e-05***
SMA	-0.1938451	0.7554621	37.06966	NA
RMA	-0.2254548	0.7588346	37.19248	9.999e-05

Confidence intervals

Method	2.5%-Intercept	97.5%-Intercept	2.5%-Slope	97.5%-Slope
OLS	-0.9878047	2.629421	0.4542434	0.8401706
MA	***-2.1518332***	***1.967823***	***0.5248301***	***0.9643632***
SMA	-2.2297868	1.387432	0.5867530	0.9726802
RMA	-2.7646433	1.630535	0.5608159	1.0297449

Eigenvalues: 0.000502658   3.545511e-05

H statistic used for computing C.I. of MA: 0.02002109

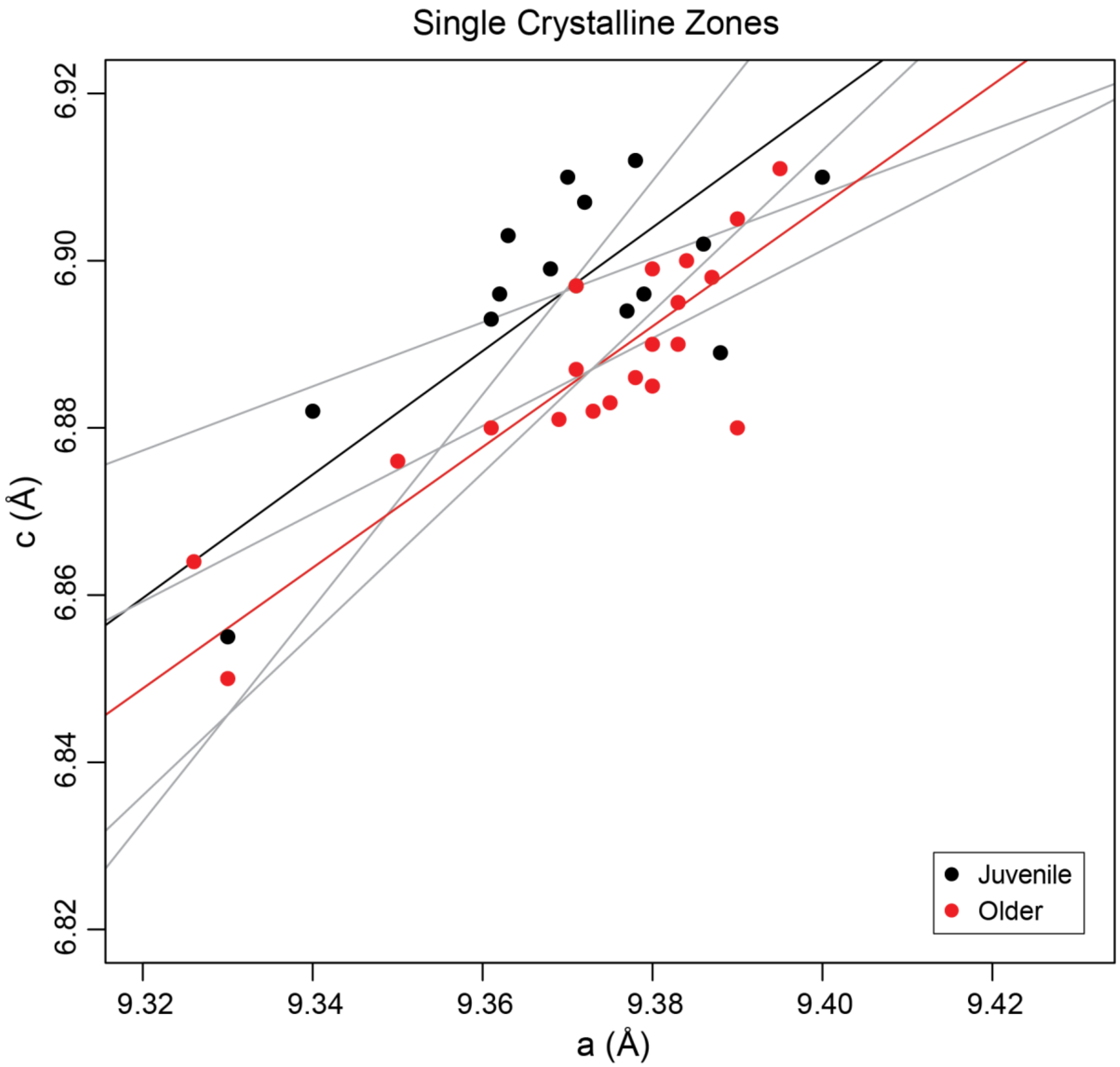

Figure S5. Major axis regressions for single crystalline zones in juvenile (black) and older (red) specimens showing 95% confidence intervals for each regression. The 95% confidence interval (C.I.) of the y-intercept value of the older specimens ($-2.158332 \leq y \leq 1.967823$) is entirely within the 95% C.I. of the y-intercept value of the juvenile specimens ($-5.071851 \leq y \leq 3.304844$, see Table S7), and even within the range of our data, the 95% C.I.s overlap significantly. As

discussed in the text, whereas the data appear visually to be separated into two regions and certainly warrant further investigation, the sample size is simply too small to separate these groups with statistical significance.

References:

1. Hughes J.M., Cameron M., Crowley K.D. 1989 Structural variations in natural F, OH, and Cl apatites. *American Mineralogist* **74**(7-8), 870-876.
2. Legendre, P. 2018 *Package 'lmodel2'*, a model II linear regression package for the programming language 'R'. available online as *cran.r-project.org/web/packages/lmodel2/lmodel.pdf*

4. 'R' Programming Calls for Model II Regression Analysis

```
#Load the required libraries
library("ggplot2")
library("lmodel2")
library("car")

#Read data from .csv file into data.frame
data <- read.csv(file.choose())

#Plot data for initial inspection using built in plot function
plot(data$a, data$c, xlab="a", ylab="c", main="All Single Crystalline")

#Plot data in ggplot2 with OLS regression line and 95% confidence window for initial inspection
ggplot(data, aes(a,c)) + geom_point() + labs(x = "a", y = "c ") + geom_smooth(method = "lm")

#Test for normal distribution of data
#Plotting Supplementary Figure S2
par(mfrow=c(3,2))
qqPlot(data$a, pch=20, cex=1.5, col="red", ylab="a ", main="All Single Crystalline")
qqPlot(data$c, pch=20, cex=1.5, col="red", ylab="c", main="All Single Crystalline")
qqPlot(data$aj, pch=20, cex=1.5, col="red", ylab="a", main="Juvenile Single Crystalline")
qqPlot(data$cj, pch=20, cex=1.5, col="red", ylab="c", main="Juvenile Single Crystalline")
qqPlot(data$am, pch=20, cex=1.5, col="red", ylab="a", main="Older Single Crystalline")
qqPlot(data$cm, pch=20, cex=1.5, col="red", ylab="c", main="Older Single Cyrstalline")

#Model II Regressions

#All Single Crystalline
AS <- lmodel2(data$c~data$a, data=data, range.y="relative", range.x="relative", nperm=10000)
print(AS)

#All Polycrystalline
AP <- lmodel2(data$cp~data$ap, data=data, range.y="relative", range.x="relative", nperm=10000)
```

```
print(AP)

#Juvenile Single Crystalline
JS <- lmodel2(data$cj~data$aj, data=data, range.y="relative", range.x="relative", nperm=10000)
print(JS)

#Older Single Crystalline
OS <- lmodel2(data$cm~data$am, data=data, range.y="relative", range.x="relative", nperm=10000)
print(OS)

#Juvenile Polycrystalline
JP <- lmodel2(data$cjp~data$ajp, data=data, range.y="relative", range.x="relative", nperm=10000)
print(JP)

#Older Polycrystalline
OP <- lmodel2(data$cmp~data$amp, data=data, range.y="relative", range.x="relative", nperm=10000)
print(OP)

#Plotting Supplementary Table S7
print(JS)
print(OS)

#Plotting Figure 4 Using all Regression Methods (Supplementary Figure S3)

#Panel A
#Plot Juvenile Single Crystalline Zones Data
plot(data$aj, data$cj, xlab = "a", ylab = "c", pch = 20, cex = 1.5, main = "Single Crystalline Zones",
xlim=c(9.32,9.43), ylim=c(6.82,6.92))

#Plot Older Single Crystalline Zones Data
points(data$am, data$cm, pch = 20, col = "red", cex = 1.5)

#Plot Legend
legend(9.38, 6.86, legend = c("OLS", "MA", "SMA", "RMA", "Juvenile", "Older"), col = c("blue", "red",
"green", "black", "black", "red"), pch = c(NA, NA, NA, NA, 20, 20), lty=c(1,1,1,1,NA,NA), ncol = 2)

#Plot Regression Lines for Juvenile Single Crystalline with Colors to Match Legend
lines(JS, "OLS", col = "blue")
lines(JS, "MA", col = "red")
lines(JS, "SMA", col = "green")
lines(JS, "RMA", col = "black")

#Plot Regression Lines for Older Single Crystalline with Colors to Match Legend
lines(OS, "OLS", col = "blue")
```

lines(OS, "MA", col = "red")
lines(OS, "SMA", col = "green")
lines(OS, "RMA", col = "black")

#Panel B

#Plot Juvenile Polycrystalline Zones Data
plot(data$ajp, data$cjp, xlab = "a", ylab = "c", pch = 20, cex = 1.5, main = "Polycrystalline Zones", xlim=c(9.32,9.43), ylim=c(6.82,6.92))

#Plot Older Polycrystalline Zones Data
points(data$amp, data$cmp, pch = 20, col = "red", cex = 1.5)

#Plot Legend
legend(9.38, 6.86, legend = c("OLS", "MA", "SMA", "RMA", "Juvenile", "Older"), col = c("blue", "red", "green", "black", "black", "red"), pch = c(NA, NA, NA, NA, 20, 20), lty=c(1,1,1,1,1,2), ncol = 2)

#Plot Regression Lines for Juvenile Single Crystalline with Colors to Match Legend
lines(JP, "OLS", col = "blue")
lines(JP, "MA", col = "red")
lines(JP, "SMA", col = "green")
lines(JP, "RMA", col = "black")

#Plot Regression Lines for Older Single Crystalline with Colors to Match Legend
lines(OP, "OLS", col = "blue")
lines(OP, "MA", col = "red")
lines(OP, "SMA", col = "green")
lines(OP, "RMA", col = "black")

#Plotting Supplementary Figure S4

par(mfrow = c(3,2))
plot(data$a, data$c, xlab = "a ", ylab = "c", pch = 20, cex = 1.5, main = "All Single Crystalline Zones", xlim=c(9.32,9.43), ylim=c(6.82,6.92))
legend(9.37, 6.86, legend = c("OLS", "MA", "SMA", "RMA"), col = c("blue", "red", "green", "black"), lty=c(1,1,1,1,NA,NA), ncol = 2, cex = 0.75)
lines(AS, "OLS", col = "blue")
lines(AS, "MA", col = "red")
lines(AS, "SMA", col = "green")
lines(AS, "RMA", col = "black")

plot(data$ap, data$cp, xlab = "a", ylab = "c", pch = 20, cex = 1.5, main = "All Polycrystalline Zones", xlim=c(9.32,9.43), ylim=c(6.82,6.92))

```
legend(9.32, 6.85, legend = c("OLS", "MA", "SMA", "RMA"), col = c("blue", "red", "green", "black"),
lty=c(1,1,1,1,NA,NA), ncol = 2, cex = 0.75)
lines(AP, "OLS", col = "blue")
lines(AP, "MA", col = "red")
lines(AP, "SMA", col = "green")
lines(AP, "RMA", col = "black")

plot(data$aj, data$cj, xlab = "a", ylab = "c", pch = 20, cex = 1.5, main = "Juvenile Single Crystalline
Zones", xlim=c(9.32,9.43), ylim=c(6.82,6.92))
legend(9.37, 6.86, legend = c("OLS", "MA", "SMA", "RMA"), col = c("blue", "red", "green", "black"),
lty=c(1,1,1,1,NA,NA), ncol = 2, cex = 0.75)
lines(JS, "OLS", col = "blue")
lines(JS, "MA", col = "red")
lines(JS, "SMA", col = "green")
lines(JS, "RMA", col = "black")

plot(data$ajp, data$cjp, xlab = "a", ylab = "c", pch = 20, cex = 1.5, main = "Juvenile Polycrystalline
Zones", xlim=c(9.32,9.43), ylim=c(6.82,6.92))
legend(9.32, 6.85, legend = c("OLS", "MA", "SMA", "RMA"), col = c("blue", "red", "green", "black"),
lty=c(1,1,1,1,NA,NA), ncol = 2, cex = 0.75)
lines(JP, "OLS", col = "blue")
lines(JP, "MA", col = "red")
lines(JP, "SMA", col = "green")
lines(JP, "RMA", col = "black")

plot(data$am, data$cm, xlab = "a", ylab = "c", col = "red", pch = 20, cex = 1.5, main = "Older Single
Crystalline Zones", xlim=c(9.32,9.43), ylim=c(6.82,6.92))
legend(9.37, 6.86, legend = c("OLS", "MA", "SMA", "RMA"), col = c("blue", "red", "green", "black"),
lty=c(1,1,1,1,NA,NA), ncol = 2, cex = 0.75)
lines(OS, "OLS", col = "blue")
lines(OS, "MA", col = "red")
lines(OS, "SMA", col = "green")
lines(OS, "RMA", col = "black")

plot(data$ajp, data$cjp, xlab = "a", ylab = "c", col = "red", pch = 20, cex = 1.5, main = "Older
Polycrystalline Zones", xlim=c(9.32,9.43), ylim=c(6.82,6.92))
legend(9.32, 6.92, legend = c("OLS", "MA", "SMA", "RMA"), col = c("blue", "red", "green", "black"),
lty=c(1,1,1,1,NA,NA), ncol = 2, cex = 0.75)
lines(OP, "OLS", col = "blue")
lines(OP, "MA", col = "red")
lines(OP, "SMA", col = "green")
lines(OP, "RMA", col = "black")
```

#Plotting Supplementary Figure S5

```
plot(data$aj, data$cj, xlab = "a", ylab = "c", pch = 20, cex = 1.5, main = "Single Crystalline Zones",
xlim=c(9.32,9.43), ylim=c(6.82,6.92))
points(data$am, data$cm, pch = 20, col = "red", cex = 1.5)
lines(JS, "MA")
lines(OS, "MA")
```
